# Modulation of Glucose Homeostasis, Metabolic Endotoxemia and Circulating Short-Chain Fatty Acids Following Multi-Species Probiotic Supplementation: Findings from a 12-Week Randomised Placebo-Controlled Trial

**DOI:** 10.3390/nu18071025

**Published:** 2026-03-24

**Authors:** George Moschonis, Pauline Dacaya, Thy T. Truong, Angela Amoruso, Marco Pane

**Affiliations:** 1 Department of Nutrition and Dietetics, Harokopio University, 70 El. Venizelou Avenue, 17671 Athens, Greece; 2 European Centre for Obesity, Harokopio University, 17671 Athens, Greece; 3 Department of Sport, Exercise and Nutrition Sciences, Discipline of Food, Nutrition and Dietetics, School Allied Health, Human Services and Sport, La Trobe University, Melbourne, VIC 3086, Australia; p.dacaya@latrobe.edu.au; 4 Proteomics and Metabolomics Platform, School of Agriculture, Biomedicine and Environment, La Trobe University, Melbourne, VIC 3086, Australia; t.truong@latrobe.edu.au; 5 Probiotical Research Srl, 28100 Novara, Italy; a.amoruso@probiotical.com (A.A.); m.pane@probiotical.com (M.P.)

**Keywords:** probiotics, glucose homeostasis, metabolic endotoxemia, incretin hormones, short-chain fatty acids

## Abstract

**Background:** Altered gut microbiota and gut-derived inflammation impair glucose regulation and promote metabolic endotoxemia, yet evidence on probiotic effects across combined glycaemic, inflammatory and short-chain fatty acid (SCFA) outcomes remains limited. This study investigated the effects of a 12-week multi-species probiotic on glucose homeostasis, incretin hormones, inflammatory biomarkers and circulating SCFAs in adults with subthreshold depression. **Methods:** In a 12-week double-blind, randomised, placebo-controlled trial, 39 adults with subthreshold depression were allocated to either a probiotic supplement containing *Limosilactobacillus fermentum* LF16, *Lacticaseibacillus rhamnosus* LR06, *Lactiplantibacillus plantarum* LP01 and Bifidobacterium longum 04 (n = 19) or placebo (n = 20). Fasting glucose, insulin, HOMA-IR, glucose-dependent insulinotropic peptide (GIP), high-sensitivity *C*-reactive protein (hs-CRP), lipopolysaccharide-binding protein (LBP), soluble CD14 (sCD14) and SCFAs were evaluated at three time points: baseline, week 6 and week 12. Between-group and treatment × time effects were analysed using general linear models. **Results:** Probiotic supplementation significantly reduced fasting glucose at 12 weeks compared with placebo (−1.8 vs. 0.1 mmol/L; *p* = 0.036). In the probiotic group, greater reductions in GIP (*p* = 0.012; *p* = 0.037), LBP (*p* < 0.001), sCD14 (*p* = 0.002; *p* = 0.001) and hs-CRP (*p* = 0.047) were also observed compared with placebo. Plasma SCFA concentrations remained largely unchanged, with no significant treatment × time interactions, except for higher valerate levels at 12 weeks in the probiotic group (*p* = 0.019). **Conclusions:** Twelve weeks of multi-species probiotic supplementation improved fasting glucose, reduced incretin and inflammatory biomarkers and attenuated metabolic endotoxemia, without alterations in circulating SCFAs. These findings support beneficial modulation of metabolic–immune pathways and highlight the promising role of probiotics to enhance glucose regulation and systemic inflammatory tone in adults with subthreshold depression.

## 1. Introduction

Alterations in gut microbial composition and metabolic function are increasingly recognised as key contributors to impaired glucose regulation, chronic low-grade inflammation and disruptions in circulating microbial metabolites such as short-chain fatty acids (SCFAs) [1]. Disturbances within the intestinal microbiota can influence host metabolic pathways through mechanisms involving enteroendocrine signalling, systemic inflammatory activation and changes in microbial fermentation products [2].

The gut microbiota exerts considerable influence over glucose homeostasis through effects on intestinal epithelial glucose transport, modulation of hepatic glucose output and interactions with incretin hormones, including glucose-dependent insulinotropic peptide (GIP) [3,4,5]. Elevated GIP concentrations have been positively correlated with insulin resistance and obesity [6], whereas reductions in fasting GIP are consistent with improved metabolic regulation and insulin sensitivity [7]. Probiotics have been shown in early human and preclinical studies to modulate glucose handling by improving epithelial integrity, reducing inflammatory stress on metabolic tissues and affecting enteroendocrine hormone secretion [8,9,10,11].

Alongside metabolic disturbances, gut-derived endotoxemia has emerged as a key contributor to chronic low-grade systemic inflammation. This process occurs when lipopolysaccharides (LPSs) translocate across a compromised intestinal barrier, triggering immune activation [12]. Circulating biomarkers such as lipopolysaccharide-binding protein (LBP) and soluble CD14 (sCD14) serve as established indicators of metabolic endotoxemia and monocyte activation [13,14]. Elevated *C*-reactive protein (CRP) further reflects the downstream inflammatory cascade initiated by LPS and Toll-like receptor 4 (TLR4) signalling [15]. These inflammatory pathways impair insulin receptor signalling and contribute to hyperglycaemia and hepatic metabolic dysregulation [16,17]. Probiotic strains, particularly from the genera *Lactobacillus* and *Bifidobacterium*, have been reported to reinforce tight-junction integrity and reduce LPS translocation [18,19].

In addition to inflammatory and metabolic pathways, SCFAs, including acetate, propionate and butyrate, greatly contribute to the regulation of epithelial barrier function, immune homeostasis and metabolic efficiency [20]. Straight-chain SCFAs are generally associated with beneficial metabolic effects, whereas branched-chain SCFAs (e.g., isobutyrate and isovalerate) reflect proteolytic fermentation and may be associated with less favourable host responses [21]. Plasma SCFA concentrations represent the integrated outcome of microbial production, epithelial absorption and host utilisation and provide insight into microbial–host metabolic interactions [22]. Yet, controlled human trials evaluating plasma SCFAs following probiotic supplementation, particularly alongside glucose and inflammatory biomarkers, remain scarce.

The present analysis represents a secondary exploratory investigation derived from a previously published double-blind randomised placebo-controlled trial that primarily evaluated the effects of this multi-species probiotic formulation on psychosocial outcomes in adults with subthreshold depression [23,24]. In addition to mental health outcomes, the parent trial included several secondary metabolic biomarkers, including fasting plasma glucose and insulin indices. Notably, probiotic supplementation resulted in a significant reduction in fasting plasma glucose concentrations, leading to normalisation of glycaemic levels within the probiotic group. This observation suggested that modulation of gut microbiota may influence metabolic pathways related to glucose regulation in this population, a finding that has rarely been reported in trials investigating probiotics primarily targeting mental health outcomes. These findings provided the rationale for the present analysis, which aims to further explore potential biological mechanisms underlying this effect by examining changes in glucose homeostasis, metabolic endotoxemia and circulating short-chain fatty acids following probiotic supplementation.

The multi-species probiotic formulation used in this study, containing *Limosilactobacillus fermentum* LF16, *Lacticaseibacillus rhamnosus* LR06, *Lactiplantibacillus plantarum* LP01 and *Bifidobacterium longum* 04, has previously been shown to modulate inflammatory and metabolic pathways in early-phase trials [23,24]. However, its effects on fasting glucose, incretin signalling, inflammatory activation and circulating SCFAs have not been evaluated together. To address this gap, the present study examined the impact of 12-week supplementation on glucose metabolism, inflammatory and immune activation markers and circulating SCFAs in adults with mild depressive symptoms. By assessing these mechanistic domains concurrently, this trial provides an integrated view of how probiotic supplementation may influence the metabolic–immune–microbial axis.

## 2. Materials and Methods

The present study builds on the findings of a 12-week, double-blind, randomised, placebo-controlled trial [23,24] conducted in compliance with the NHMRC National Statement on Ethical Conduct in Human Research (2007), the Note for Guidance on Good Clinical Practice (CPMP/ICH-135/95), and the CONSORT statement. The Human Research Ethics Committee of La Trobe University (HEC21032; approval date: 15 March 2021) approved all procedures involving human subjects, and all volunteers provided written informed consent. The trial protocol was registered with the Australia New Zealand Clinical Trials Registry (ACTRN12621000675820). The complete methodological framework, including recruitment strategies, sample size considerations, detailed participant assessments and safety procedures, has been described extensively in two previously published research articles arising from the same trial [23,24].

### 2.1. Study Participants

Participants were recruited in Melbourne, Australia, using online platforms, flyers and a commercial recruitment service. Screening included administration of the Structured Clinical Interview for DSM-5 to identify individuals presenting with subthreshold depressive symptoms while excluding those with major psychiatric disorders, chronic inflammatory or gastrointestinal diseases, recent antibiotic or probiotic use, or other conditions likely to interfere with metabolic outcomes. Eligible adults aged 18–65 years with a BMI ≥ 18.5 kg/m^2^ were invited to participate and provided written consent before enrolment. Thirty-nine participants (13 males and 26 females) were ultimately randomised, with 19 allocated to the probiotic arm and 20 to the placebo.

### 2.2. Intervention

Randomisation occurred in a 1:1 ratio using block randomisation procedures overseen by an independent investigator not involved in data collection or analysis. The probiotic and placebo capsules were indistinguishable in appearance and packaging, ensuring complete blinding of both participants and research staff. Each participant was instructed to consume one capsule daily for 12 weeks. The probiotic supplement contained a lyophilised mixture of active bacteria strains (1 × 10^9^ live cells per strain) from the following genera: *Limosilactobacillus fermentum* LF16 (DSM26956), *Lacticaseibacillus rhamnosus* LR06 (DSM21981), *Lactiplantibacillus plantarum* LP01 (LMG P-21021) and *Bifidobacterium longum* 04 (DSM23233) [23,24]. The probiotic is commercially available as Biome Lift ^TM^ in Australia and as Bifizen ^TM^ in the USA and Europe. The placebo capsules were formulated with the same excipient blend (i.e., glyceryl palmitostearate (E471), silicon dioxide, maltodextrin, hypromellose, and titanium dioxide) as the probiotic capsules, omitting only the active bacterial strains [23,24]. Analysis via flow cytometry (ISO 19344, 2015: IDF 232: 2015) [25] and CFU plate count (Internal Method 014-06) confirmed a cell count of >4 × 10^9^ Active Fluorescent Units (AFUs)/g of the probiotic product at batch release (Biolab srl, Novara, Italy) [23,24]. The study sponsor (Biome Australia Trading Pty Ltd., Collingwood, VIC Australia) provided probiotic and placebo products packaged in identical, shelf-stable blister packs.

### 2.3. Study Visits

Study visits were conducted at baseline, 6 weeks, and 12 weeks. All data were collected by trained research personnel using standardised procedures and operating protocols.

### 2.4. Socio-Demographics

Participant data were collected during the screening phase and baseline visit encompassing socio-demographic characteristics (e.g., education, employment, ethnicity, and country of birth), health status (e.g., weight and height), lifestyle habits (e.g., smoking) and relevant medical history [23,24].

### 2.5. Dietary Intake, Physical Activity and Anthropometrics

Dietary intake was assessed using 3-day food diaries collected, and physical activity (PA) was recorded using the Active Australia Survey (AAS) [26] at each time point to aid interpretation of potential confounders. These behavioural measures were not controlled but were reviewed to confirm the absence of systematic group differences that could bias outcomes.

Anthropometric measurements were obtained at baseline, week 6, and week 12. A column scale and stadiometer (Seca 703, Hamburg, Germany) were used to measure body weight and standing height to the closest 0.1 kg and 0.1 cm, respectively, where participants wore light clothing and were barefoot [23,24]. Body mass index (BMI) was calculated using Quetelet’s equation (weight (kg)/height (m)^2^).

### 2.6. Blood Collection and Processing

Collection of venous blood was performed by a trained phlebotomist at baseline, week 6, and week 12 after a 10–12 h overnight fast. Samples were centrifuged (Hettich Rotina 420R, Tuttlingen, Germany) at 2739 rcf for 10 min at 4 °C, where the resulting plasma and/or serum were aliquoted into portions of 500 µL and stored at –80 °C prior to analysis [23,24].

### 2.7. Laboratory Analyses

#### 2.7.1. Glycaemic and Insulin Parameters

As previously reported [23,24], an ELISA (KAQ1251, Thermo Fisher Scientific, Waltham, MA USA; sensitivity 0.17 µIU/mL; assay range 5.1–250 µIU/mL) was used to measure serum insulin, an ELISA (EIAGLUC, Thermo Fisher Scientific, USA; sensitivity 0.413 mg/dL; assay range 0.5–32 mg/dL) was used to assess glucose, and the Homeostatic Model Assessment (HOMA-IR), using the equation fasting insulin (µIU/mL) × fasting glucose (mmol/L)/22.5, was used to estimate insulin resistance.

#### 2.7.2. Incretin Hormones

Serum glucagon-like peptide-1 (GLP-1) was measured using a comparable sandwich ELISA (FineTest^®^ Human GLP-1 Kit; Cat. EH1053-HS, Wuhan, China), employing an anti-GLP-1 capture surface, biotin-conjugated detection antibody, HRP–streptavidin amplification, TMB substrate, and absorbance measurement at 450 nm. Standard curves were generated using eight calibrators (1.563–100 pg/mL; sensitivity 0.938 pg/mL). Although all assays were run in duplicate according to the manufacturer’s protocol, GLP-1 concentrations were below the assay’s detection limit and are therefore not reported further.

Serum glucose-dependent insulinotropic peptide (GIP) was quantified using a sandwich ELISA (FineTest^®^ Human GIP Kit; Cat. EH0562). Standards and diluted serum samples were added to a 96-well plate pre-coated with an anti-GIP capture antibody, followed by a biotin-labelled detection antibody and horseradish peroxidase (HRP)–streptavidin. Colour development with 3,3′,5,5′-tetramethylbenzidine (TMB) was terminated by the addition of an acidic solution. Absorbance was subsequently determined at 450 nm. Concentrations were interpolated from a seven-point standard curve (46.875–3000 pg/mL; sensitivity 28.125 pg/mL). All samples were analysed in duplicate.

#### 2.7.3. Inflammatory Peptides

Serum high-sensitivity *C*-reactive protein (hs-CRP) was measured using ELISA (BMS288INST, Thermo Fisher Scientific, USA; sensitivity 3.0 pg/mL; assay range 78–5000 pg/mL). All hs-CRP values were screened to identify potential acute infections; no participant recorded hs-CRP > 10 mg/L. Serum lipopolysaccharide-binding protein (LBP) was determined using a sandwich ELISA (FineTest^®^ Human LBP Kit; Cat. EH1560). After incubation of standards and diluted serum samples in anti-LBP-coated wells, a biotin-labelled detection antibody and HRP–streptavidin were applied. Signals were generated using the TMB substrate, stopped with an acidic solution, and read at 450 nm. LBP concentrations were calculated from an eight-point calibration curve (31.25–2000 pg/mL; sensitivity 18.75 pg/mL). All analyses were performed in duplicate. Serum-soluble CD14 (sCD14) was quantified using a sandwich ELISA (Huanke Bio^®^ Human sCD14 Kit; Cat. HK320-0212, Beijing, China) involving anti-sCD14-coated wells, a biotin-conjugated detection antibody, HRP–streptavidin, and the TMB substrate. Absorbance at 450 nm was converted to concentrations using a standard curve spanning the manufacturer-specified analytical range. Samples were assayed in duplicate to ensure reproducibility.

#### 2.7.4. Quantification of Short-Chain Fatty Acids by GC-MS

Plasma SCFAs (C1–C7, formic to heptanoic acids) were quantified using gas chromatography–mass spectrometry (GC-MS) following the methods of Furuhashi et al. (2019) [27] with minor modifications. Plasma samples (150 µL) were extracted using a liquid–liquid procedure with hexanoic acid-D3 as the internal standard. After centrifugation, the aqueous fraction was derivatised using isobutyl chloroformate and extracted into hexane. Calibration standards (0–50 mg/L), QC standards (10 mg/L), and laboratory blanks underwent the same preparation.

GC-MS analysis was performed using a Trace 1300 Series GC coupled to a TSQ 8000 Evo triple quadrupole MS (Thermo Fisher Scientific). A VF-5ms capillary column (30 m × 0.25 mm, 0.5 µm) was used with helium as the carrier gas at 1 mL/min. The oven was programmed from 35 °C (5 min) to 310 °C at 10 °C/min (1 min hold). Electron-impact ionisation (70 eV) was applied, and acquisition used full-scan mode (*m*/*z* 35–400).

Data acquisition and processing were performed using Xcalibur and Thermo FreeStyle, with spectral matching using the NIST 2020 library (≥70% match). QC standards were used to monitor analytical stability and retention time shifts, and blanks were included to detect contamination.

### 2.8. Sample Size

Sample size estimation was based on expected differences in mean BDI-II score reductions from pre- to post-intervention between the probiotic and placebo groups [28]. Data from 40 participants (20 per group) was calculated to provide at least 80% power (α = 0.05, two-tailed) to detect a minimal important 4.2-point mean difference between groups. Post hoc power assessment indicated that the final sample size was adequate for the majority of the examined secondary outcome biomarkers, with the exception of short-chain fatty acids (SCFAs), for which the study was slightly underpowered.

### 2.9. Statistical Analysis

Statistical analyses were performed using SPSS for Windows (version 25.0). Analyses followed an intention-to-treat approach. Missing outcome data were handled using multiple imputation so that all randomised participants could be retained in the analyses wherever possible. The distribution of continuous variables was assessed prior to modelling, and normally distributed variables are presented as mean (standard deviation).

For each biochemical outcome, longitudinal changes across the three study time points (baseline, 6 weeks, and 12 weeks) were examined using repeated-measures general linear models, with time specified as the within-subject factor and treatment group (probiotic vs. placebo) as the between-subject factor. The main parameter of interest was the treatment × time interaction, which was used to determine whether the trajectory of change over follow-up differed between the two intervention arms. Main effects for treatment and time were also examined. When significant main or interaction effects were identified, Bonferroni-adjusted post hoc comparisons were used to explore pairwise differences between time points and between groups at each assessment.

All models were adjusted for baseline educational level and dietary covariates identified from the parent trial dataset as relevant potential confounders, specifically changes in dietary energy intake from baseline to weeks 6 and 12, changes in dietary protein intake to week 12, and mean dietary fibre intake at week 6, as applicable to the respective outcome models [23,24]. All reported *p*-values were two-tailed, while the level of statistical significance was set at *p* < 0.05.

## 3. Results

Table 1 presents the baseline socio-demographic and anthropometric characteristics of participants allocated to the placebo (n = 20) and probiotic (n = 19) groups. The two groups were generally well-matched across all continuous variables, with no significant differences observed for age, weight, height, or BMI. Mean age was comparable between groups (41.8 ± 11.4 vs. 42.9 ± 15.8 years; *p* = 0.804), and similar patterns were seen for weight, height, and BMI, all of which showed non-significant between-group differences. The distribution of BMI categories (i.e., normal weight, overweight, and obesity) was also similar across treatment arms (*p* = 0.413). Gender distribution did not differ significantly between groups, with females comprising the majority in both arms. Employment status, place of birth, and region of residence were likewise comparable, with most participants born in Australia or New Zealand and residing in urban areas. A small proportion of individuals in the probiotic group reported living in rural regions, although this difference did not reach statistical significance (*p* = 0.064). Educational attainment was the only variable showing a statistically significant difference between groups (*p* = 0.024). Participants in the probiotic arm were more likely to hold postgraduate qualifications, whereas those in the placebo group more frequently reported certificate/diploma-level or trade qualifications. Overall, apart from differences in education level, the placebo and probiotic groups were broadly similar at baseline in terms of demographic and anthropometric characteristics.

Table 2 shows that baseline glycaemic and inflammatory markers were comparable between groups. Across the duration of the intervention, the probiotic group demonstrated a clear improvement in fasting glucose, with reductions of −0.9 mmol/L at 6 weeks and −1.8 mmol/L at 12 weeks, while the placebo group showed no change. This difference between groups became statistically significant at 12 weeks (*p* = 0.036). Serum insulin and HOMA-IR showed small, non-significant decreases in both groups, although the probiotic group displayed a favourable trend toward improved insulin sensitivity by week 12. Differences were also observed for systemic inflammation, since hs-CRP decreased in the probiotic group at both 6 and 12 weeks (i.e., by −822.6 ng/mL, 95% CI: −1528.2 to −116.9; by −954.6 ng/mL, 95% CI: −1914.9 to 5.69, respectively), whereas levels increased in the placebo arm. The treatment × time interaction at 12 weeks for hs-CRP was also statistically significant (*p* = 0.047), highlighting a reduction in low-grade inflammation among participants receiving the probiotic compared to the placebo.

**Figure 1:**
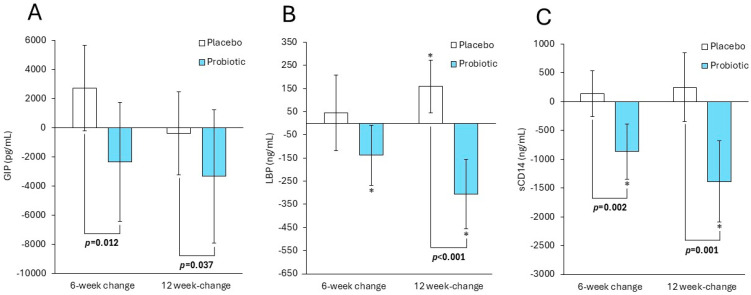
Mean changes (95% CI) of incretin and inflammation-related peptides in people diagnosed with subthreshold depression receiving either a probiotic food supplement (n = 19) or a placebo (n = 19), from baseline to weeks 6 and 12. These changes refer to (**A**) serum glucose-dependent insulinotropic peptide (GIP, pg/mL), (**B**) serum lipopolysaccharide-binding protein (LBP, ng/mL), and (**C**) serum-soluble CD14 (sCD14, ng/mL). * Indicates significant (*p* < 0.05) within-group changes from baseline to follow-up. *p*-values indicate the differences in the changes from baseline to 6- and 12-week follow-ups between the two treatment groups (i.e., treatment × time interaction effect).

Figure 1 summarises the changes observed in incretin and inflammation-related peptides across the 12-week intervention. Fasting serum GIP concentrations remained relatively stable in the placebo group, whereas the probiotic group showed sustained reductions at both 6 and 12 weeks. These decreases were significantly greater than those observed in the placebo arm from baseline to weeks 6 and 12, respectively (*p* = 0.012; *p* = 0.037), indicating a consistent treatment × time interaction. For LBP, values in the placebo group increased progressively over time, while the probiotic group exhibited significant reductions of −138.6 ng/mL (95% CI: −267.7 to −9.6) at 6 weeks and −306.1 ng/mL (95% CI: −455 to −157.3) at 12 weeks. Between-group differences/treatment × time interaction effects were also statistically significant at 12 weeks (*p* < 0.001), reflecting a marked divergence in trajectories. A comparable trend was identified for sCD14, with the probiotic group demonstrating significant declines at both follow-up points (i.e., by −865.7 ng/mL, 95% CI: −1342.9 to −388.6 at 6 weeks; −1386.7 ng/mL, 95% CI: −2091.9 to −681.5 at 12 weeks), contrasting with modest increases in the placebo group. These between-group differences/treatment × time interaction effects were significant at both 6 weeks (*p* = 0.002) and 12 weeks (*p* = 0.001). Collectively, these results show that participants receiving the probiotic experienced reductions in fasting GIP, LBP, and sCD14 over time, whereas those in the placebo group tended to show stable or increasing levels.

Table 3 presents changes in plasma SCFAs over the 12-week intervention. Overall, none of the individual SCFAs showed significant treatment × time interaction effects. Concentrations of acetate and propionate, the main straight-chain SCFAs, remained largely stable in both groups, with small non-significant fluctuations at 6 and 12 weeks. Formate levels increased modestly in both groups at 6 weeks and then partially returned toward baseline by week 12, without meaningful between-group differences. Branched-chain SCFAs (isobutyrate and isovalerate), which reflect proteolytic fermentation, showed small decreases in both groups over time, again with no evidence of a differential probiotic effect. Valerate concentrations declined slightly in the placebo group but showed a modest rise in the probiotic arm, resulting in a significant between-group difference in mean valerate levels at week 12 (*p* = 0.019), although changes from baseline were not statistically different. Collectively, these results indicate that probiotic supplementation did not produce substantial alterations in circulating SCFAs, with overall profiles remaining stable across the intervention period.

The distribution of circulating biomarker concentrations at each study time point (baseline, 6 weeks, and 12 weeks) for the probiotic and placebo groups is presented in Supplementary Figures S1–S3. These violin plots illustrate the variability and central tendency of glycaemic control indices (Supplementary Figure S1), inflammatory and metabolic biomarkers (Supplementary Figure S2), and circulating short-chain fatty acids (SCFAs; Supplementary Figure S3) across treatment arms. Overall, the distributions indicate broadly comparable baseline profiles between groups and visually demonstrate the divergence in trajectories over time for selected glycaemic and inflammatory biomarkers in participants receiving the probiotic.

Changes in biomarker concentrations from baseline to follow-up are further illustrated in Supplementary Figures S4–S6, which depict within-participant changes from baseline to 6 weeks (T1–T2) and from baseline to 12 weeks (T1–T3), stratified by treatment arm. As shown in Supplementary Figure S4, participants receiving the probiotic tended to exhibit greater reductions in fasting plasma glucose and related glycaemic indices compared with the placebo, particularly at 12 weeks, whereas changes in insulin and HOMA-IR were modest and overlapped between groups. Consistent with the mean changes reported in Table 2 and Figure 1, Supplementary Figure S5 demonstrates more pronounced downward shifts in inflammatory and metabolic endotoxemia markers, including hs-CRP, GIP, LBP and sCD14, in the probiotic group compared with the placebo, especially over the full 12-week intervention period. In contrast, Supplementary Figure S6 shows largely overlapping distributions of changes in circulating SCFAs between treatment arms, indicating minimal differential effects of probiotic supplementation on systemic SCFA concentrations.

Collectively, these Supplementary Figures provide a complementary visual representation of the central tendencies and inter-individual variability underlying the primary statistical analyses, supporting the observed improvements in glycaemic control and inflammatory biomarkers following probiotic supplementation, alongside relatively stable SCFA profiles.

## 4. Discussion

The findings of the current trial demonstrate that a 12-week supplementation with a multi-species probiotic, containing *Limosilactobacillus fermentum* LF16, *Lacticaseibacillus rhamnosus* LR06, *Lactiplantibacillus plantarum* LP01 and *Bifidobacterium longum* 04, produced favourable changes across several mechanistic pathways central to metabolic and inflammatory regulation. The most notable change was the substantially reduced fasting glucose observed in the probiotic group compared with the placebo. This reduction occurred without a parallel rise in circulating insulin, suggesting that the improvement in glycaemic status was unlikely to be driven by increased insulin secretion and more plausibly reflected enhanced insulin sensitivity or improved regulation of endogenous glucose production [29]. The concurrent decrease in fasting GIP provides further support for this interpretation, as elevated fasting GIP is often observed in states of insulin resistance and impaired incretin responsiveness [3]. Rather than indicating suppressed incretin action, the reduction in fasting GIP may reflect a normalisation of enteroendocrine function in a context of improved metabolic regulation [30]. Together, these observations point toward a metabolic environment that requires less incretin stimulus to maintain glycaemic control, consistent with enhanced efficiency of glucose handling.

The relevance of these findings is particularly noteworthy in the context of subthreshold depression, which represents a common but often under-recognised condition characterised by mild depressive symptoms and increased risk of progression to major depressive disorder [31]. Beyond psychological manifestations, accumulating evidence indicates that individuals with subthreshold depressive symptoms frequently present with subtle metabolic and inflammatory disturbances, including alterations in glucose regulation and low-grade systemic inflammation [32,33]. These biological alterations are increasingly recognised as important contributors to the gut–brain–metabolic axis linking metabolic dysfunction with mood disorders [34,35,36]. Within this context, the improvements observed in glycaemic regulation and markers of metabolic endotoxemia following probiotic supplementation may reflect broader physiological processes that could contribute to improved metabolic resilience in individuals experiencing early-stage depressive symptoms.

The improvements observed in glucose metabolism were also accompanied by substantial changes in markers of metabolic endotoxemia and systemic inflammation. Participants receiving the probiotic exhibited clear reductions in circulating LBP and sCD14, two biomarkers that may reflect distinct stages of the host immune response to gut-derived LPS [12]. Reductions in LBP may be indicative of reduced exposure to bacterial endotoxins or improved epithelial barrier integrity, while decreases in sCD14 suggest diminished monocyte activation [13,14]. The simultaneous reduction in hs-CRP further supports a broad attenuation of inflammatory tone [37]. The consistency of these findings across three complementary biomarkers suggests that the probiotic intervention may provide potentially meaningful alterations of the gut–immune interface by reducing the translocation or immunogenic impact of microbial products from the intestine into the circulation. Given the well-established role of endotoxemia in impairing insulin signalling and contributing to metabolic dysregulation [17], these anti-inflammatory shifts likely further contributed to the observed improvements in glucose homeostasis.

Although the SCFAs did not exhibit statistically significant treatment–time interactions, the overall pattern of change supports an interpretation of microbial metabolic stability in the probiotic group. Acetate and propionate, the predominant straight-chain SCFAs associated with favourable metabolic and immunological effects [38], remained stable or increased slightly in the probiotic group, while the placebo group showed either no improvement or modest reductions. In contrast, branched-chain SCFAs such as isobutyrate and isovalerate, which are considered indicators of protein fermentation and have been linked to less favourable metabolic conditions [39], decreased or remained stable in both groups and did not demonstrate any adverse elevations in the probiotic group. These findings imply that the probiotic did not promote excessive proteolytic fermentation, which can yield metabolites detrimental to epithelial and metabolic health. The small rise in valerate observed in the probiotic group, while not statistically significant in change from baseline, may reflect subtle shifts toward increased cross-feeding interactions within the microbial community [40]. Importantly, the SCFA profile did not show any indication of dysregulated fermentation or disproportionate rises in circulating SCFAs, which has been proposed as a potential concern in certain metabolic contexts [41].

Overall, the integration of glucose, inflammatory and SCFA findings provides a possible coherent mechanistic pathway in which probiotic supplementation supports a shift toward improved metabolic efficiency, reduced inflammatory activation and balanced microbial metabolic activity. Reductions in endotoxemia markers are particularly meaningful because they can represent an upstream regulatory point capable of influencing both metabolic and inflammatory pathways [17]. The improvement in glucose homeostasis is consistent with the concept that lowering LPS-mediated inflammatory signalling alleviates the inhibitory pressure placed on insulin pathways [42]. Similarly, the generally stable or modestly favourable SCFA profile is compatible with improved epithelial barrier function and reduced LPS translocation, two processes central to maintaining metabolic and inflammatory homeostasis [39].

Key strengths of the current study lie in its stringent, randomised, double-blind methodology, the concurrent measurement of multiple mechanistic biomarkers, and the use of a probiotic formulation with strains known for their metabolic and immune modulatory potential. Nevertheless, limitations should also be acknowledged, including the modest sample size and the absence of stool microbiome sequencing, which would have provided additional insight into the microbial shifts underlying the observed plasma biomarker changes. Plasma SCFAs also represent systemic rather than luminal concentrations and therefore reflect both production and utilisation, complicating direct interpretation of microbial fermentation patterns [38,39].

## 5. Conclusions

Despite these limitations, the results of this study contribute meaningful evidence that multi-species probiotic supplementation, containing *Limosilactobacillus fermentum* LF16, *Lacticaseibacillus rhamnosus* LR06, *Lactiplantibacillus plantarum* LP01 and *Bifidobacterium longum* 04, can beneficially modulate glucose regulation, reduce biomarkers of gut-derived inflammatory activation and maintain a favourable systemic SCFA profile. These interrelated findings align with the broader conceptual model in which the gut microbiota exerts important regulatory effects across metabolic and inflammatory systems. Future research incorporating microbial sequencing, controlled dietary conditions and larger sample sizes will help clarify the specific microbial pathways through which probiotics exert these effects and the populations most likely to benefit from such interventions.

## Figures and Tables

**Table 1:** Baseline anthropometric and socio-demographic distribution among treatment groups.

	Placebo (n = 20)	Probiotic (n = 19)	*p*-Value *
	**Mean (SD)**	**Mean (SD)**	
Age (years)	41.8 (11.4)	42.9 (15.8)	0.804
BMI (kg/m^2^)	28.8 (6.6)	27.6 (6.4)	0.568
	**n (%)**	**n (%)**	
Weight status			0.413
Normal weight	6 (30.0)	9 (47.4)	
Overweight	7 (35.0)	3 (15.8)	
Obese	7 (35.0)	7 (36.8)	
Gender			0.365
Males	8 (40.0)	5 (26.3)	
Females	12 (60.0)	14 (73.7)	
Education			**0.024**
Apprentice/trade	2 (10.0)	0 (0.0)	
Secondary school	0 (0.0)	4 (21.1)	
Certificate/diploma	7 (35.0)	2 (10.5)	
Bachelor’s degree	8 (40.0)	6 (31.6)	
Master’s/PhD	3 (15.0) *	7 (36.9) *	
Employment			0.443
Unemployed	3 (15.0)	1 (5.3)	
Casual employment	4 (20.0)	4 (21.1)	
Part-time employment	5 (25.0)	2 (10.5)	
Full-time employment	8 (40.0)	11 (57.9)	
Retired	0 (0.0)	1 (5.3)	
Birth place			0.763
Australia/New Zealand	16 (80.0)	15 (78.9)	
Europe	1 (5.0)	2 (10.5)	
Asia	3 (15.0)	2 (10.5)	
Region of residence			0.064
Urban	20 (100.0)	16 (84.2)	
Rural	0 (0.0)	3 (15.8)	

Data are presented as mean (standard deviation: SD) or as frequencies (n) and percentages (%). * *p* values were derived using Student’s *t*-test for continuous variables and the chi-square test for categorical variables. Bold values indicate statistically significant differences (*p* < 0.05) between treatment arms at baseline [23,24].

**Table 2:** Changes in biochemical markers of glycaemic profile and inflammation in people diagnosed with subthreshold depression receiving either a probiotic food supplement (n = 19) or a placebo (n = 19), from baseline to 6 and 12 weeks of intervention.

	Baseline	6-Week Follow-Up	6-Week Change	12-Week Follow-Up	12-Week Change
	Mean (SD)	Mean (SD)	Mean (95% CI)	Mean (SD)	Mean (95% CI)
**Fasting Plasma** **Glucose (mmol/L)**					
Placebo	6.3 (4.3)	6.3 (3.9)	−0.01 (−0.78; 0.77)	6.4 (4.8)	0.1 (−1.3; 1.5)
Probiotic	6.2 (3.3)	5.3 (3.2)	**−0.9 (−1.5; −0.2)**	4.4 (2.9)	**−1.8 (−2.8; −0.7)**
*p*-value ^†^	0.918	0.419	0.070 *	0.347	0.036 *
**Serum Insulin** **(μIU/mL)**					
Placebo	9.7 (8.0)	9.5 (6.0)	−0.2 (−4.8; 4.5)	9.5 (6.8)	−0.2 (−4.4; 4.0)
Probiotic	9.1 (5.2)	8.5 (6.2)	−0.6 (−4.6; 3.3)	8.6 (7.6)	−0.5 (−5.5; 4.4)
*p*-value ^†^	0.821	0.785	0.715 *	0.542	0.821 *
**HOMA-IR**					
Placebo	3.2 (3.6)	2.6 (2.0)	−0.6 (−2.0; 0.9)	2.7 (3.0)	−0.4 (−1.6; 0.7)
Probiotic	2.6 (2.1)	2.6 (3.4)	0.01 (−1.3; 1.3)	2.0 (2.6)	−0.6 (−1.9; 0.8)
*p*-value ^†^	0.477	0.905	0.585 *	0.744	0.854 *
**hs-CRP (ng/mL)**					
Placebo	6720.8 (1190.7)	6682.5 (1600.5)	−38.3 (−459.8; 383.2)	7286.2 (1205.8)	565.4 (−297.4; 1428.3)
Probiotic	6931.0 (1517.2)	6108.4 (1540.0)	**−822.6 (−1528.2; −116.9)**	5976.4 (1408.3)	−954.6 (−1914.9; 5.69)
*p*-value ^†^	0.850	0.157	0.054 *	**0.003**	0.047 *

*: Treatment × time interaction effect; ^†^: Between-group differences in mean values at baseline, 6 weeks and 12 weeks, as well as in 6- and 12-week changes from baseline (treatment effect). Adjustments were made for study participants’ educational level, changes in dietary energy intake from baseline to 6 weeks and from baseline to 12 weeks, changes in dietary protein intake from baseline to 12 weeks and mean dietary fibre intake at 6 weeks. SD, standard deviation; CI, confidence interval; HOMA-IR, Homeostatic Model Assessment for Insulin Resistance; hs-CRP, high-sensitivity *C*-reactive protein. Results in bold indicate statistical significance (*p* < 0.05).

**Table 3:** Changes in plasma concentrations of short-chain fatty acids in individuals with a subthreshold depression diagnosis receiving either a probiotic food supplement (n = 19) or a placebo (n = 19), from baseline to 6 and 12 weeks of intervention.

	Baseline	6-Week Follow-Up	6-Week Change	12-Week Follow-Up	12-Week Change
	Mean (SD)	Mean (SD)	Mean (95% CI)	Mean (SD)	Mean (95% CI)
**Short-Chain Fatty Acids** **Acetate (ng/μL)**					
Placebo	2.40 (1.22)	2.51 (1.51)	0.11 (−1.47; 1.69)	2.12 (1.95)	−0.28 (−1.62; 1.05)
Probiotic	2.48 (1.07)	2.46 (3.08)	−0.12 (−1.60; 1.57)	2.73 (1.45)	0.25 (−1.09; 1.59)
*p*-value ^†^	0.866	0.955	0.826 *	0.310	0.778 *
**Formate (ng/μL)**					
Placebo	2.41 (2.51)	3.52 (4.35)	1.11 (−2.40; 4.62)	2.74 (2.40)	0.34 (−2.18; 2.85)
Probiotic	2.70 (1.93)	4.28 (5.07)	1.58 (−1.93; 5.09)	3.38 (3.49)	0.67 (−1.84; 3.19)
*p*-value ^†^	0.718	0.672	0.554 *	0.544	0.965 *
**Propionate (ng/μL)**					
Placebo	0.44 (0.45)	0.35 (0.30)	−0.09 (−0.40; 0.23)	0.41 (0.30)	−0.03 (−0.37; 0.32)
Probiotic	0.39 (0.39)	0.34 (0.25)	−0.05 (−0.37; 2.63)	0.52 (0.37)	0.13 (−0.22; 0.47)
*p*-value ^†^	0.772	0.893	0.865 *	0.410	0.713 *
**Isobutyrate (ng/μL)**					
Placebo	0.60 (0.56)	0.41 (0.31)	−0.19 (−0.49; 0.11)	0.38 (0.28)	−0.22 (−0.47; 0.04)
Probiotic	0.58 (0.18)	0.41 (0.30)	−0.17 (−0.47; 0.13)	0.36 (0.30)	−0.22 (−0.48; 0.04)
*p*-value ^†^	0.876	0.982	0.938 *	0.827	0.983 *
**Isovalerate (ng/μL)**					
Placebo	1.08 (0.92)	0.93 (0.61)	−0.15 (−0.74; 0.44)	0.83 (0.38)	−0.24 (−0.72; 0.24)
Probiotic	1.00 (0.55)	1.07 (0.91)	0.07 (−0.52; 0.66)	1.07 (0.78)	0.07 (−0.41; 0.55)
*p*-value ^†^	0.766	0.598	0.805 *	0.266	0.550 *
**Valerate (ng/μL)**					
Placebo	0.60 (0.53)	0.50 (0.34)	−0.10 (−0.50; 0.29)	**0.29 (0.26)**	−0.31 (−0.66; 0.04)
Probiotic	0.55 (0.47)	0.70 (0.74)	0.15 (−0.24; 0.55)	**0.72 (0.68)**	0.17 (−0.18; 0.52)
*p*-value ^†^	0.727	0.358	0.225 *	**0.019**	0.086 *

*: Treatment × time interaction effect; ^†^: Between-group differences in mean values at baseline, 6 weeks and 12 weeks, as well as in 6- and 12-week changes from baseline (treatment effect). Adjustments were made for study participants’ educational level, changes in dietary energy intake from baseline to weeks 6 and 12, changes in dietary protein intake from baseline to week 12 and mean dietary fibre intake at 6 weeks. SD, standard deviation; CI, confidence interval. Results in bold indicate statistical significance (*p* < 0.05).

## Data Availability

The data supporting the findings of this study are derived from a human clinical trial and are subject to ethical restrictions. The study was approved by the La Trobe University Human Research Ethics Committee, which does not permit the public deposition of individual-level participant data, even in de-identified form, due to privacy and confidentiality considerations. In accordance with the ethics approval and participant consent, de-identified data may be made available upon reasonable request to the Principal Investigator and corresponding author, Professor George Moschonis, subject to appropriate confidentiality and data-use agreements.

## Supplementary Materials

The following supporting information can be downloaded at https://www.mdpi.com/article/10.3390/nu18071025/s1: Figure S1: Violin plots depicting the distribution of glycaemic control biomarkers, including fasting plasma glucose, insulin, and HOMA-IR, at baseline (T1), 6-week (T2), and 12-week (T3) follow-up time points in study participants receiving either the probiotic food supplement (n = 19) or the placebo (n = 19). Individual participant values are overlaid, horizontal lines indicate median values, and violin widths represent the kernel density of the data; Figure S2: Violin plots illustrating the distribution of inflammatory and metabolic biomarkers, including high-sensitivity *C*-reactive pro-tein (hsCRP), glucose-dependent insulinotropic peptide (GIP), lipopolysaccharide-binding protein (LBP), and soluble CD14 (sCD14), at baseline (T1), 6-week (T2), and 12-week (T3) follow-up in par-ticipants receiving either the probiotic food supplement (n = 19) or the placebo (n = 19). Individual data points are shown, median values are indicated by horizontal lines, and violin widths reflect the density of observations; Figure S3: Violin plots showing the distribution of circulating short-chain fatty acids, including acetate, formate, propionate, isobutyrate, isovalerate, and valerate, measured at baseline (T1), 6-week (T2), and 12-week (T3) follow-up in participants assigned to the probiotic food supplement (n = 19) or placebo (n = 19). Individual participant values are overlaid, horizontal lines indicate median values, and violin widths represent the kernel density of the data; Figure S4: Violin plots showing changes (Δ) in glycaemic control indices, including fasting plasma glucose (FPG), insulin, and HOMA-IR, from baseline to 6 weeks (T1–T2) and from baseline to 12 weeks (T1–T3) in participants receiving either the probiotic food supplement (n = 19) or the placebo (n = 19). Individual participant values are overlaid, horizontal lines indicate median changes, and violin widths represent the kernel density of the data. Positive and negative values denote increases and decreases from baseline, respectively; Figure S5: Violin plots illustrating changes (Δ) in inflammatory and metabolic biomarkers, including high-sensitivity *C*-reactive protein (hsCRP), glucose-dependent insulinotropic peptide (GIP), lipopolysaccharide-binding protein (LBP), and soluble CD14 (sCD14), from baseline to 6 weeks (T1–T2) and from baseline to 12 weeks (T1–T3) in participants assigned to the probiotic food supplement (n = 19) or placebo (n = 19). Individual data points are shown, median values are indicated by horizontal lines, and violin widths reflect the distribution density of the observed changes; Figure S6: Violin plots depicting changes (Δ) in circulating short-chain fatty acids (SCFAs), including acetate, formate, propionate, isobutyrate, isovalerate, and valerate, from baseline to 6 weeks (T1–T2) and from baseline to 12 weeks (T1–T3) in participants receiving the probiotic food supplement (n = 19) or placebo (n = 19). Individual participant values are overlaid, horizontal lines indicate median changes, and violin widths represent the kernel density of the data.
