# Supplementary material for: Modulation of Glucose Homeostasis, Metabolic Endotoxemia and Circulating Short-Chain Fatty Acids Following Multi-Species Probiotic Supplementation: Findings from a 12-Week Randomised Placebo-Controlled Trial
Source: Nutrients. 2026 Mar 24;18(7):1025. doi: 10.3390/nu18071025 (PMC13074660; doi:10.3390/nu18071025)
Supplement: Supplementary file 1 [file nutrients-18-01025-s001.zip › Supplementary Figures 1 to 6 Titles and Captions.pdf]

Supplementary Figure 1. Violin plots depicting the distribution of glycaemic control biomarkers, including fasting plasma glucose, insulin, and HOMA-IR, at baseline (T1), 6-week (T2), and 12-week (T3) follow-up time points in study participants receiving either the probiotic food supplement (n = 19) or the placebo (n = 19). Individual participant values are overlaid, horizontal lines indicate median values, and violin widths represent the kernel density of the data.

Supplementary Figure 2. Violin plots illustrating the distribution of inflammatory and metabolic biomarkers, including high-sensitivity C-reactive protein (hsCRP), glucose-dependent insulinotropic peptide (GIP), lipopolysaccharide-binding protein (LBP), and soluble CD14 (sCD14), at baseline (T1), 6-week (T2), and 12-week (T3) follow-up in participants receiving either the probiotic food supplement (n = 19) or the placebo (n = 19). Individual data points are shown, median values are indicated by horizontal lines, and violin widths reflect the density of observations.

Supplementary Figure 3. Violin plots showing the distribution of circulating short-chain fatty acids, including acetate, formate, propionate, isobutyrate, isovalerate, and valerate, measured at baseline (T1), 6-week (T2), and 12-week (T3) follow-up in participants assigned to the probiotic food supplement (n = 19) or placebo (n = 19). Individual participant values are overlaid, horizontal lines indicate median values, and violin widths represent the kernel density of the data.

Supplementary Figure 4. Violin plots showing changes (Δ) in glycaemic control indices, including fasting plasma glucose (FPG), insulin, and HOMA-IR, from baseline to 6 weeks (T1–T2) and from baseline to 12 weeks (T1–T3) in participants receiving either the probiotic food supplement (n = 19) or the placebo (n = 19). Individual participant values are overlaid, horizontal lines indicate median changes, and violin widths represent the kernel density of the data. Positive and negative values denote increases and decreases from baseline, respectively.

Supplementary Figure 5. Violin plots illustrating changes (Δ) in inflammatory and metabolic biomarkers, including high-sensitivity C-reactive protein (hsCRP), glucose-dependent insulinotropic peptide (GIP), lipopolysaccharide-binding protein (LBP), and soluble CD14 (sCD14), from baseline to 6 weeks (T1–T2) and from baseline to 12 weeks (T1–T3) in participants assigned to the probiotic food supplement (n = 19) or placebo (n = 19). Individual data points are shown, median values are indicated by horizontal lines, and violin widths reflect the distribution density of the observed changes.

Supplementary Figure 6. Violin plots depicting changes (Δ) in circulating short-chain fatty acids (SCFAs), including acetate, formate, propionate, isobutyrate, isovalerate, and valerate, from baseline to 6 weeks (T1–T2) and from baseline to 12 weeks (T1–T3) in participants receiving the probiotic food supplement (n = 19) or placebo (n = 19). Individual participant values are overlaid, horizontal lines indicate median changes, and violin widths represent the kernel density of the data.
