# Supplementary figures and images for: Modulation of Glucose Homeostasis, Metabolic Endotoxemia and Circulating Short-Chain Fatty Acids Following Multi-Species Probiotic Supplementation: Findings from a 12-Week Randomised Placebo-Controlled Trial
Source: Nutrients. 2026 Mar 24;18(7):1025. doi: 10.3390/nu18071025 (PMC13074660; doi:10.3390/nu18071025)

## Glycaemic control indices

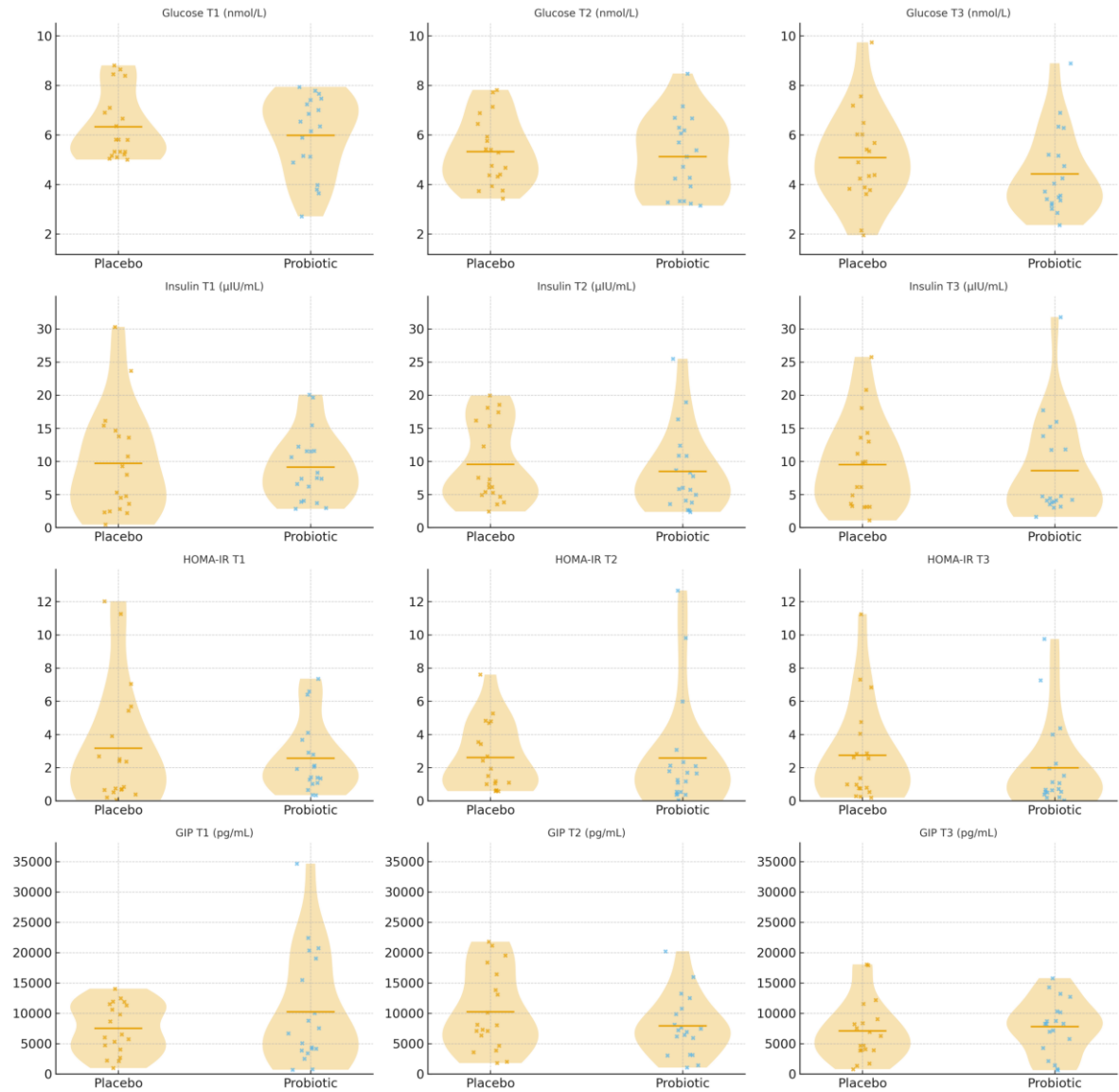

## Inflammatory biomarkers

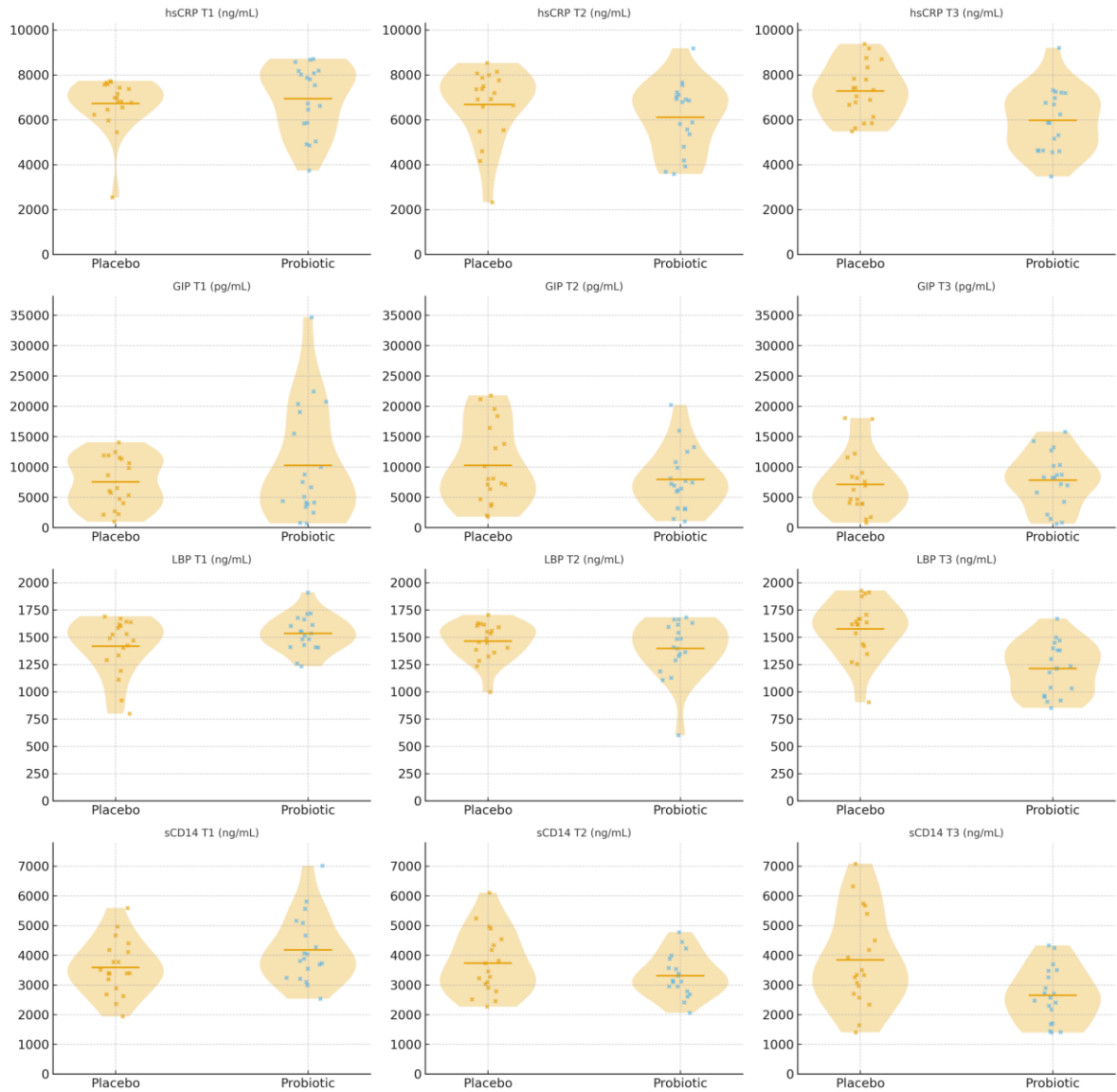

# SCFA profiles

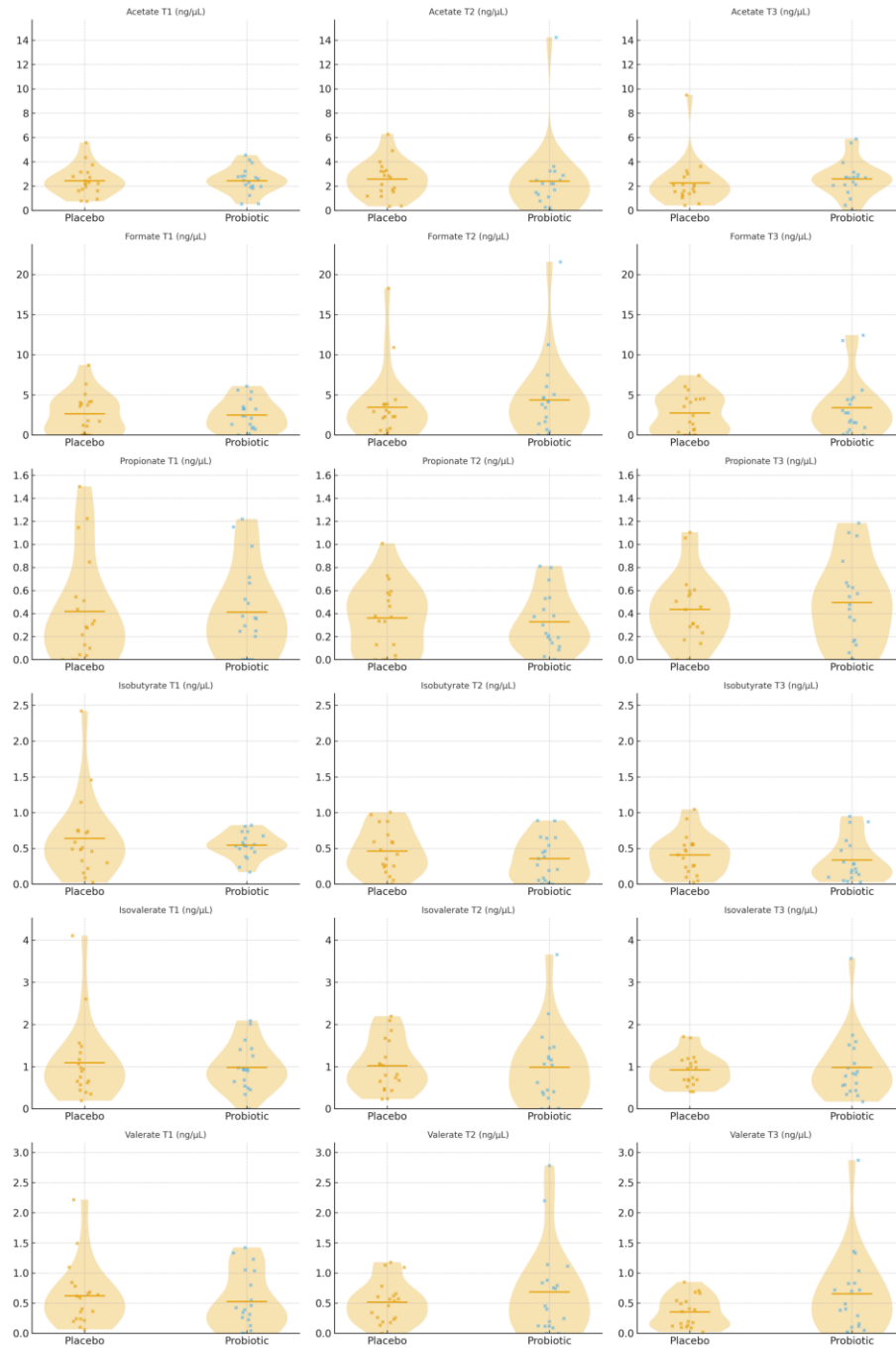

Supplement: Supplementary file 1 [file nutrients-18-01025-s001.zip › Supplementary Figures 1 to 3 Violin plots showing the distribution of circulating biomarkers concentrations per time point and treatment arm.pdf]

# Glycaemic control indices

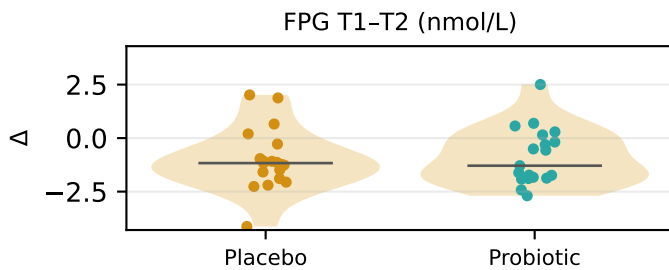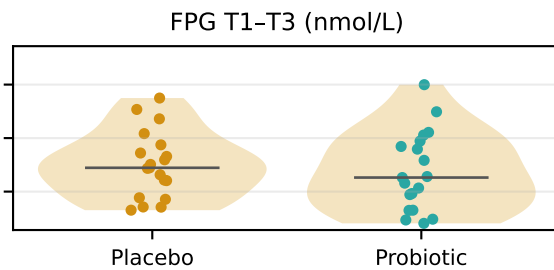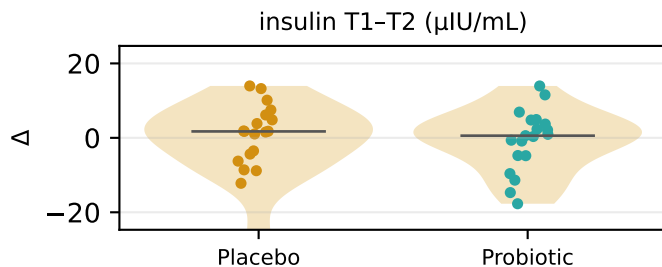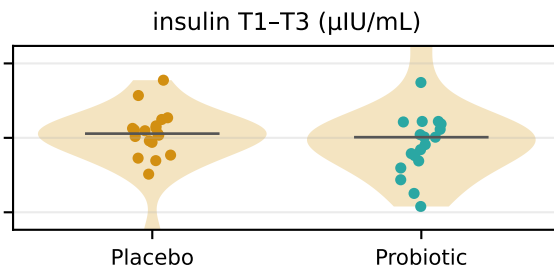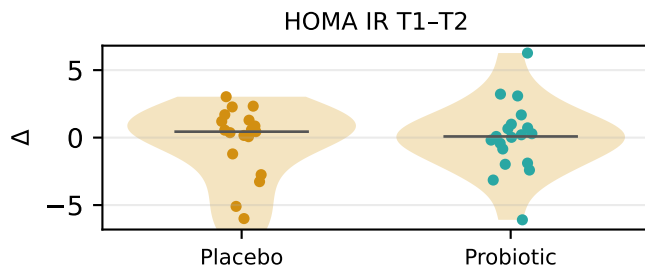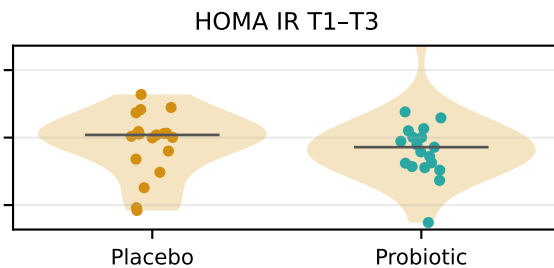

# Inflammatory biomarkers

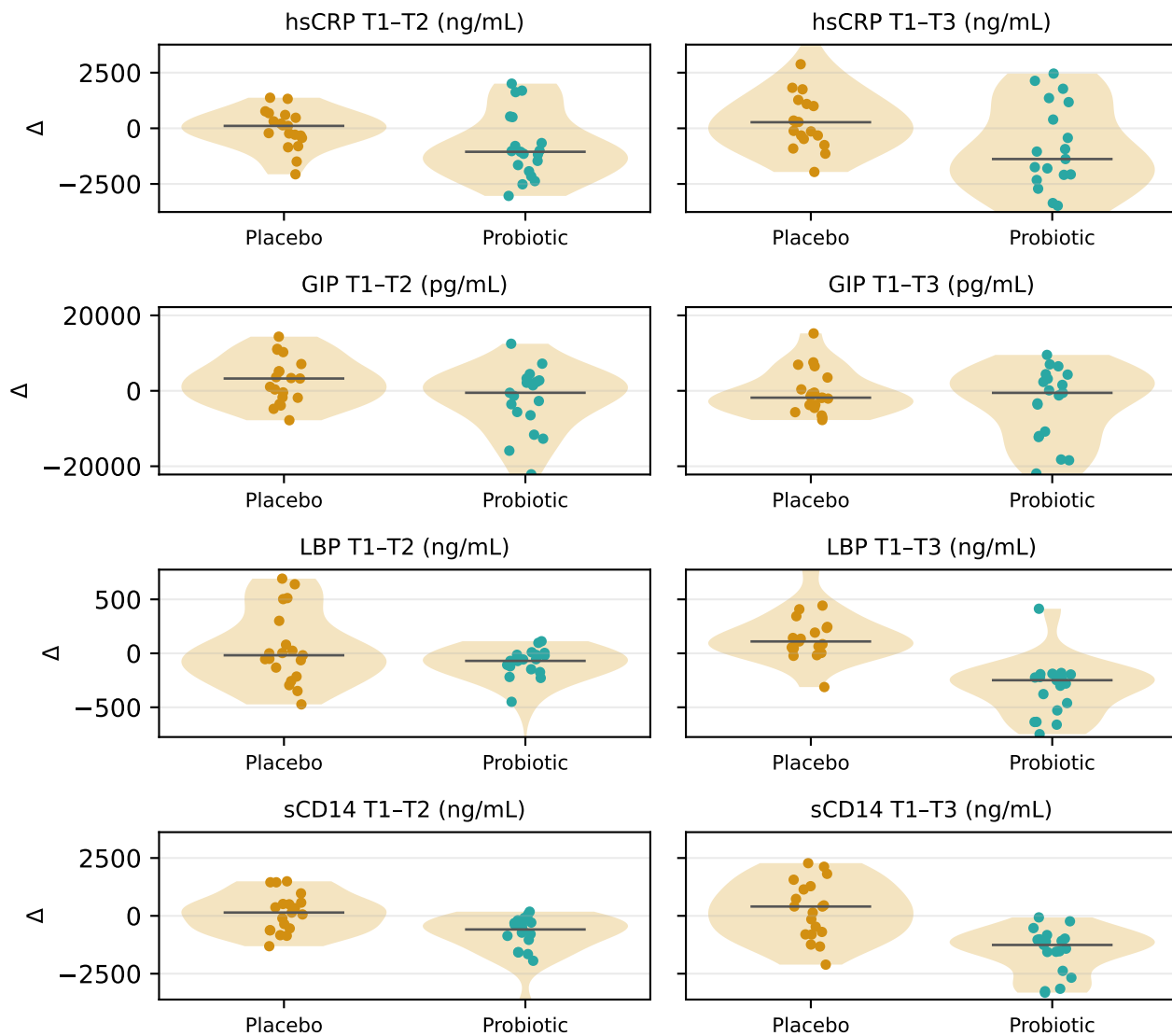

# SCFA profiles

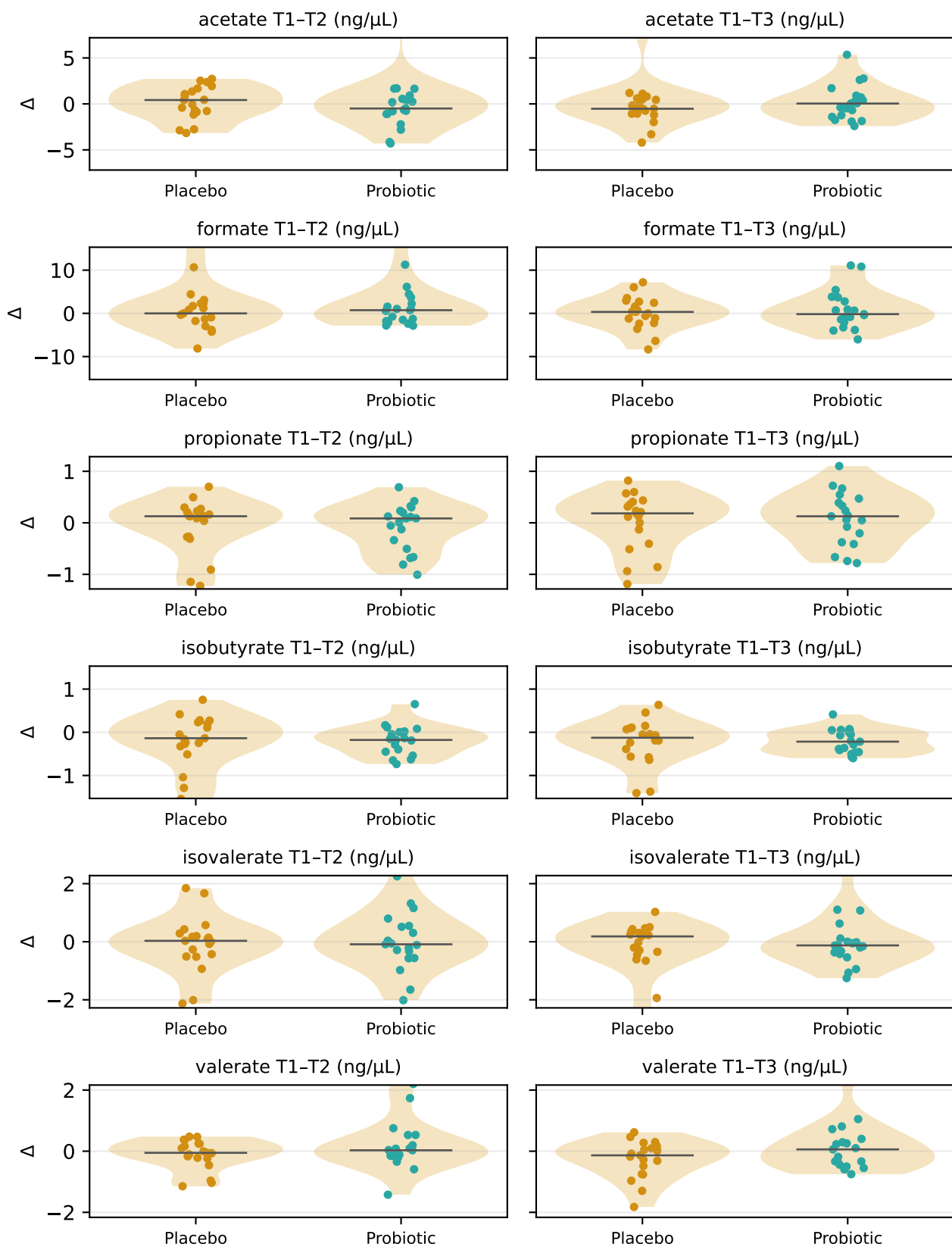

Supplement: Supplementary file 1 [file nutrients-18-01025-s001.zip › Supplementary Figures 4 to 6 Violin plots showing the distribution in the changes of circulating biomarkers concentrations per time point and treatment arm.pdf]
